# Effect of mono-guanidine-like derivatives on platelet aggregation and tumour cell induced platelet aggregation†

**DOI:** 10.1039/d4md00793j

**Published:** 2025-01-31

**Authors:** Nadhim Kamil Hante, Aaron P. Keogh, Yanni Huang, Tanya Kapoor, Harriet Bennett-Lenane, Eleanor Walsh, Isabel Rozas, Carlos Medina, Maria Jose Santos-Martinez

**Affiliations:** a School of Pharmacy & Pharmaceutical Sciences, Trinity College Dublin, Panoz Institute, The University of Dublin D02 PN40 Dublin Ireland carlos.medina@tcd.ie santosmm@tcd.ie; b Faculty of Pharmacy, University of Kufa Al-Najaf Iraq; c School of Chemistry, Trinity Biomedical Sciences Institute, Trinity College Dublin, The University of Dublin 152-160 Pearse Street D02 R590 Dublin Ireland rozasi@tcd.ie; d School of Medicine, Trinity Biomedical Sciences Institute, Trinity College Dublin, The University of Dublin 152-160 Pearse Street D02 R590 Dublin Ireland

## Abstract

Antiplatelet agents are the cornerstone for the treatment and prevention of cardiovascular diseases. However, they can induce severe side effects such as gastrointestinal bleeding. The main aim of this study is to determine the effect that novel guanidine-based derivatives exert on platelet aggregation. From a screening, in collaboration with the Psychoactive Drug Screening Project service of several compounds from our in-house library of α2-adrenoceptors' ligands, four compounds showed high to medium affinity towards α2C-adrenoceptors and H2 histamine receptors. Based on the structure of these compounds, another two in-house α2-adrenoceptors' ligands were also selected. The effect of the six compounds on platelet aggregation was investigated by light transmission aggregometry and optical microscopy. Flow cytometry was used to analyse their effect on platelet activation by measuring the expression of GPIIb/IIIa and P-selectin platelet receptors. Finally, the potential effect of those compounds on tumour cell-induced platelet aggregation was studied on three cancer cell lines from different origins using optical microscopy. We found that three of these compounds, with very good affinity towards H2 histamine receptors, significantly inhibited platelet aggregation, induced by both ADP and collagen, at the highest concentrations tested, and that tumour cell-induced platelet aggregation was also modulated by these derivatives. Our findings suggest that these aryl guanidine-like systems have an antiplatelet effect that could be also beneficial to reduce tumour cell–platelet interactions.

## Abstract 2

Antiplatelet agents are the cornerstone for the treatment and prevention of cardiovascular diseases.

## Graphical abstract

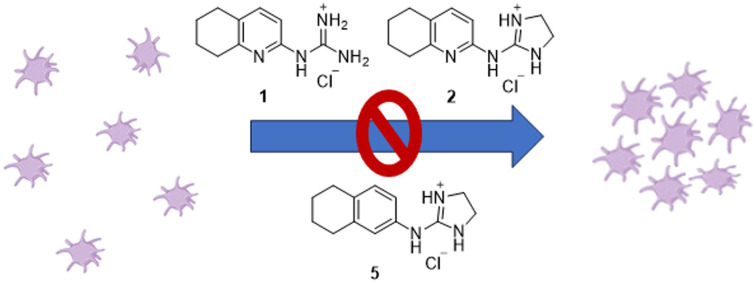

## Introduction

Platelets play an important role in the pathophysiology of cardiovascular diseases; however, they are also implicated in other pathological processes such as inflammation, infection, and cancer.^1,2^ Several drugs, including acetylsalicylic acid (aspirin), are known to impair platelet function and are used as antiplatelet agents to prevent and treat thrombotic events.^3^ However, antiplatelet therapy has been associated with undesirable side effects, such as gastro-duodenal ulcer complications. In fact, it is estimated that the incidence of gastrointestinal bleeding with the use of low-dose aspirin is 0.48–3.64 cases per 1000 persons per year.^4^ Therefore, there is still appetite for the development of new therapeutic strategies.

Platelet activation comprises multiple, complex, and imbricated signalling processes that involve rearrangement of the platelet cytoskeleton, platelet shape change, granule secretion, mobilization of calcium and the recruitment of more platelets for the formation of the definitive platelet plug.^5^ Platelet aggregation is mainly mediated by the platelet integrin receptor GPIIb/IIIa, which allows the binding of fibrinogen to the receptors of adjacent platelets.^6^ In addition, P-selectin, which is one of the most predictable markers of platelet activation, mediates the initial platelet–leukocyte tethering and triggers leukocyte activation interacting with specific carbohydrate ligands on leukocytes called P-selectin glycoprotein ligand-1 (PSGL-1).^7^ Clear evidence also suggests that tumour cells can induce platelet activation and aggregation, a phenomenon commonly known as tumour cell-induced platelet aggregation (TCIPA).^8–11^ In addition, it has been previously shown that induced thrombocytopenia is correlated with a reduction in the number of metastases in experimental models of cancer metastasis^12^ and high platelet counts are also commonly cited to predict poor outcomes in cancer patients.^13^ Therefore, the development of novel therapeutic compounds, which could target tumour cell–platelet interactions, without the side effects of the agents currently used, may form the basis for a safer and more successful approach for the management of cancer-associated thrombosis and prevention of tumour metastasis.

Several studies have described the involvement of different neurotransmitters in platelet aggregation. Some of these studies have confirmed the presence of many binding sites on platelets which possess α2-adrenergic receptor (α2-AR) characteristics.^14–16^ The aggregatory effect of noradrenaline (NA) and adrenaline (AD) on platelets is not mediated by ADP, thromboxane A2 or other agonists released by activated platelets. NA and AD seem to perform the primary function of platelet aggregation, potentiation of stimulus induced aggregation and secretion along with the inhibition of adenylate cyclase by activation of the α2-ARs.^17^ Hence, it seems that the aggregatory response of platelets to AD is induced by selective α2-AR agonists (*e.g.* clonidine or lofexidine),^18,19^ whereas it can be inhibited by using α2-AR antagonists. Modification in platelet aggregation and the number of α2-AR in the brain of depressive patients are also in agreement with the change in α2-AR density.^20^

Additionally, it has been demonstrated that histamine, which is widely distributed in body tissues and has a variety of physiological functions,^21,22^ enhances platelet aggregation induced by several pro-aggregatory agonists in a concentration-dependent manner.^23,24^ Histamine H2 receptor antagonists are well known to exert antiplatelet effects.^25–27^ Additionally, famotidine, ranitidine and cimetidine, which are known H2 receptor antagonists, have also been reported to inhibit platelet aggregation induced by collagen and ADP in platelet rich plasma (PRP) at concentrations of 1.4–1.5 mM.^28^

Guanidine-like derivatives are used extensively in many drug classes, including cardiovascular drugs, antibiotics and antidiabetics as well as antihistamines such as cimetidine and famotidine for the treatment of gastrointestinal reflux.^29^ Searching for antidepressants, Rozas' group has been working in guanidine-like derivatives for more than 20 years identifying many α2-AR ligands with agonist and antagonist activities.^30–33^ The guanidine group may be responsible for the main chemical properties and biological activity of these drugs, including a potential antiplatelet effect.^34^ In fact, platelet dysfunction is commonly observed as an undesirable side effect of many drugs such as antidepressants, antihistamines, antibiotics, antipsychotics and chemotherapeutic agents.^35^

The aim of this study is two-fold; firstly, considering the relationship between α2-AR binding and platelet aggregation, the effect that guanidine-based α2-AR agonists and antagonists, previously prepared in Rozas' group, has on platelet function was evaluated. Secondly, the potential effect on TCIPA of those compounds that showed anti-aggregation activity was assessed. The compounds considered are phenyl and pyridinyl guanidines and 2-aminoimidazolines previously reported by Rozas' group as α2-AR's ligands;^30–33^ however, considering the complexity and all possible activation mechanisms of platelet aggregation mentioned above, and that other targets (*e.g.* H2 histamine receptors) may be of importance, it seemed necessary to screen the potential engagement of these compounds to other potentially relevant biogenic amine targets. For that reason, a selection of α2-AR agonists and antagonists with different aromatic cores attached to a guanidine-like group from the ‘in-house’ Rozas' library was first submitted to the NIMH Psychoactive Drug Screening Programme (PDSP)^36,37^ to be screened for their potential affinity not only for (nor)adrenaline and histamine receptors, but also for other biogenic amine receptors and transporters.

**Fig. 1:**
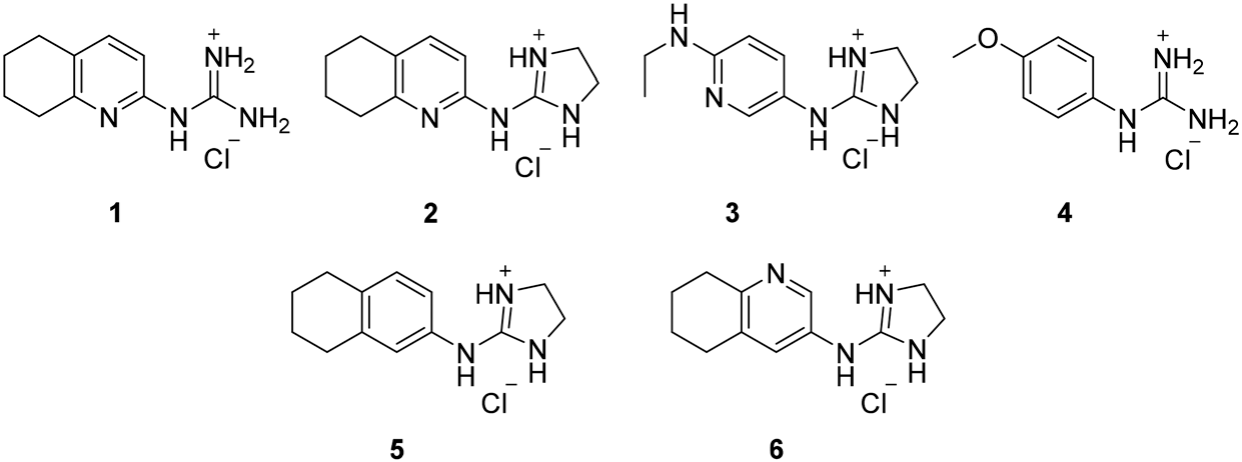
Structures of compounds chosen from Rozas' ‘in-house’ library of α2-adrenoceptors' ligands: 1 to 4 selected from the PDSP screening for their affinity towards α2-AR and H2 histamine receptors and 5 and 6 selected for their structural similarity to 1–4 and good α2-AR functional binding.

From this PDSP screening, four compounds showing high to medium affinity towards α2C-AR and H2 histamine receptors were selected (1–3 (ref. 33) and 4,^32^Fig. 1), and considering their structure, other two in-house analogues (compounds 5 (ref. 30) and 6,^33^Fig. 1), known to be ligands of α2-ARs from previous functional studies, were also chosen for this study.

## Results

### Screening for relevant receptors at the PDSP

Samples of previously reported mono-guanidine-like aryl α2-AR ligands were submitted to the PDSP to explore their binding to different receptors and transporters of relevant biogenic neurotransmitters. These compounds were chosen based on the good results obtained in previous α2-AR functional assays^30–33^ as well as the diversity in their aromatic cores. First, it was determined whether any members of the screening samples exhibited affinities not only for adrenergic (α1A-, α2A-, α2C-, and β2-ARs) and histamine (H2 and H3) receptors, but also for dopaminergic (D1-like: D1 and D5, and D2-like: D2 and D4), serotonergic (5-HT1A, 5-HT1B, 5-HT1D, 5-HT1E, 5-HT2A, 5-HT2B, 5-HT2C, 5-HT3) and sigma (σ1 and σ2) receptors. Other targets examined included monoamine transporters (DAT, NET and SERT). From the results obtained,^38^ four compounds (1–4, Fig. 1) showed interesting affinity towards α2-ARs and histamine H2 receptors and, for that reason, they were moved forward to further assessment of their potential effect on platelet aggregation.

Preparation of compounds 1 to 4, some of which had been previously identified by us as α2-AR antagonists (1–3),^33^ was carried out according to the procedures previously reported by our group in the literature (compounds 1–3 from ref. 33, and compound 4 from ref. 32) and their purity was assessed to be >95% by HPLC prior to their biological evaluations.

None of the four compounds selected showed any affinity towards any of the monoamine transporters tested (DAT, NET or SERT). This is important because it limits the poly-pharmacology of these derivatives. Regarding dopamine receptors, these compounds did not show affinity towards either the D2 or the D5 subtypes and only compound 1 displayed low affinity for the D1 and D4 receptors (involved in Parkinson's disease and attention deficit hyperactivity disorder, respectively) (Table 1). In the case of the screening for serotoninergic receptors, none of the four compounds exhibited affinity for the 5-HT1D, 5-HT1E and 5-HT3 receptors' subtypes. However, compound 1 exhibited low affinity towards 5-HT1B and 5-HT2A and medium to high affinity towards 5-HT2B and 5-HT2C, compound 2 had medium affinity for 5-HT2B and 5-HT2C, compound 3 showed low affinity for 5-HT1A, 5-HT2B and 5-HT2C and compound 4 exhibited very low affinity for 5-HT2B (Table 1). Receptors 5-HT1A and 5-HT2B seem to be involved in anxiety whereas 5-HT2A is implicated in schizophrenia. Antidepressant and antipsychotic drugs can exhibit antiplatelet activity as side effects.^35,39,40^ Concerning the sigma receptors (σ1 and σ2), which are extensively expressed in the central nervous system and are the targets for psychotropic drugs such as benzomorphans, cocaine, methamphetamine or methylenedioxymethamphetamine (MDMA),^41^ only compounds 1 and 2 exhibited low affinity towards σ1, and compound 1 also showed medium affinity for σ2 (Table 1).

**Table 1:** Values of affinity constants (*K*_i_, nM) calculated by the PSDP for several biogenic receptors: dopamine D1 and D4 receptors, serotonin 5-HT1A, 5-HT1A, 5-HT1A, 5-HT1A and 5-HT1A, sigma σ1 and σ2, histamine H2 receptors and α2A and α2C adrenoceptors (see details in the ESI†)

Receptors	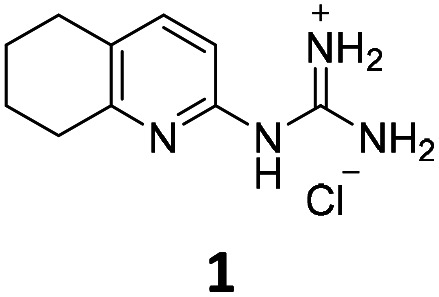	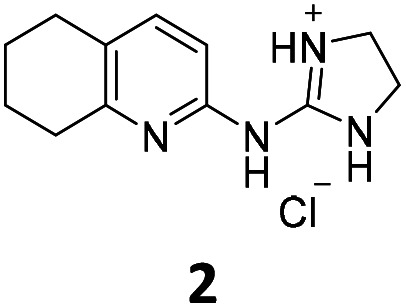	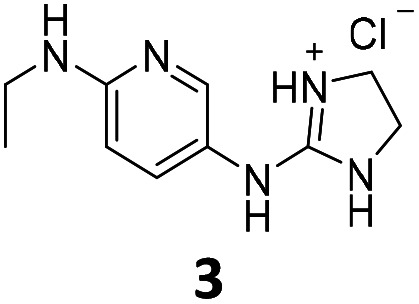	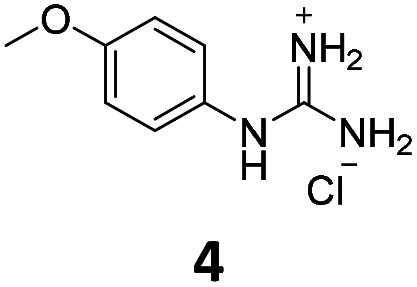
D1	968	—	—	—
D4	2042	—	—	—
5-HT1A	—	—	772	—
5-HT1B	792	—	—	—
5-HT2A	960	—	—	—
5-HT2B	36.6	40	1320	2773
5-HT2C	14	63	129	—
σ1	266	179	—	—
σ2	547	—	—	—
H2	80	202	5320	—
α2A	—	635	—	—
α2C	2059	2339	3657	1832

These compounds were also tested for their affinity for histamine receptors and none of them showed affinity for the H3 subtype; however, compounds 1, 2 and 3 had medium to low affinity for H2 receptors, which are targets for the gastroprotective effect (Table 1). As mentioned previously, antihistaminic drugs have been related to the antiplatelet effect and these results justify the proposed antiaggregant study for compounds 1, 2 and 3.

Finally, this set of compounds was explored for their binding to adrenergic receptors. None of the four compounds exhibited affinity for the β-ARs (related to hypertension or asthma) or for the α1A-AR subtype (related to benign prostatic hyperplasia). However, the four compounds showed low affinity for the α2C-ARs and compound 4 also had low affinity for the α2A-AR subtype (Table 1). As mentioned in the introduction, antidepressants target these receptor subtypes and treatment with these drugs has been associated with the anti-platelet effect. Thus, these four compounds were assessed for their potential antiplatelet activity.

### Additional compounds selected

**Fig. 2:**
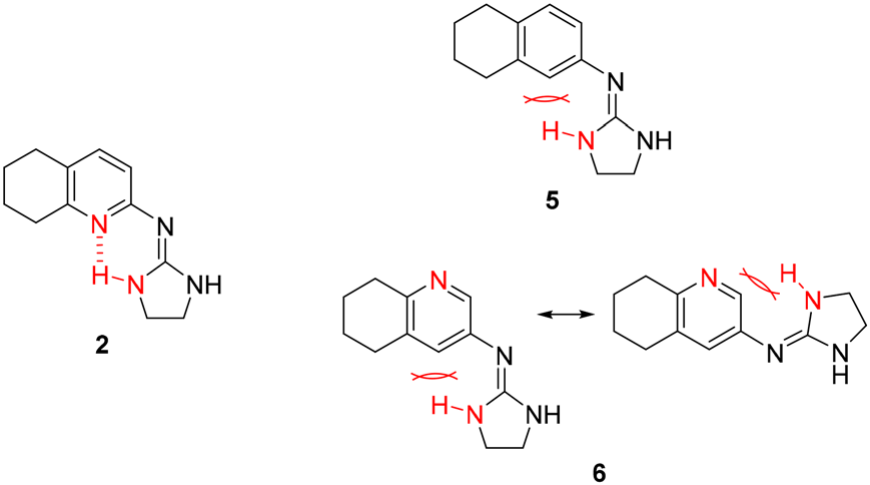
Structures of compound 2 where an IMHB can be established and analogues 5 and 6 in which no IMHB can be formed.

Previous work within our group discovered that pyridin-2-yl-guanidinium and 2-aminoimidazolinium derivatives could adopt a coplanar arrangement between the pyridine ring and the planar cationic system due to an intramolecular hydrogen bond (IMHB) between the pyridine's N atom and H from the guanidinium-like system.^33,42,43^ This coplanar conformation locking the orientation of the guanidinium results in a more limited interaction with the target, whereas a freely rotating guanidinium could facilitate other interactions within the binding site. With this idea in mind, two additional compounds were chosen from the ‘in-house’ Rozas' library as analogues of compound 2 in which no IMHB could be formed: compound 5 (Fig. 1) because it is a phenyl derivative and compound 6 (Fig. 1) because the pyridine N atom is in the *meta* position with respect to the 2-aminoimidazolinium system (Fig. 2).

By avoiding the possibility of forming an IMHB while still having similar cationic groups to compound 2, we hoped to understand whether an unlocked guanidinium/2-aminoimidazolinium group could favor the interaction with the target(s), thus affecting the anti-platelet activity. Furthermore, these compounds had shown good affinity for α2-ARs in previous functional studies performed by our group (5 (ref. 30) and 6 (ref. 33)).

### Effect of the compounds selected on platelet aggregation

**Fig. 3:**
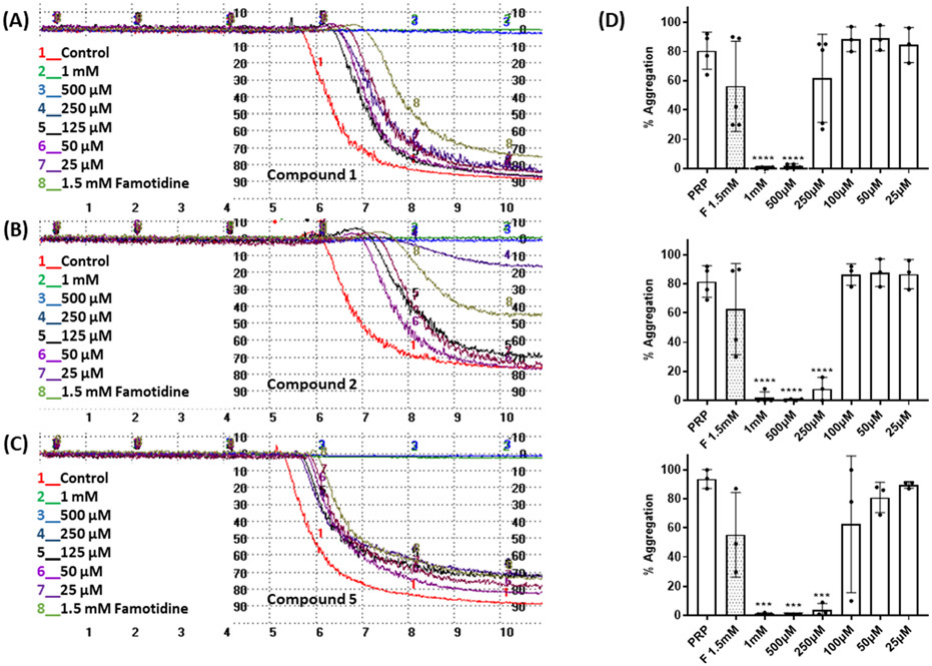
Effect of compounds 1, 2 and 5 on platelet aggregation induced by collagen (2 μg mL^−L^). Representative traces from light transmission aggregometry showing the effect of 1.5 mM famotidine and (A) 1, (B) 2 and (C) 5 on collagen-induced platelet aggregation at various concentrations (25 μM to 1 mM). Untreated platelet rich plasma (PRP) stimulated by collagen was used as the control for platelet aggregation. (D) Quantitative data as per (A)–(C) above. Data are presented as mean ± SD; *n* ≥ 3. One way ANOVA and Dunnett's multiple comparison test; ****P < 0.0001; ***P < 0.001 *vs.* control (PRP in the absence of the compounds). Famotidine-F.

**Fig. 4:**
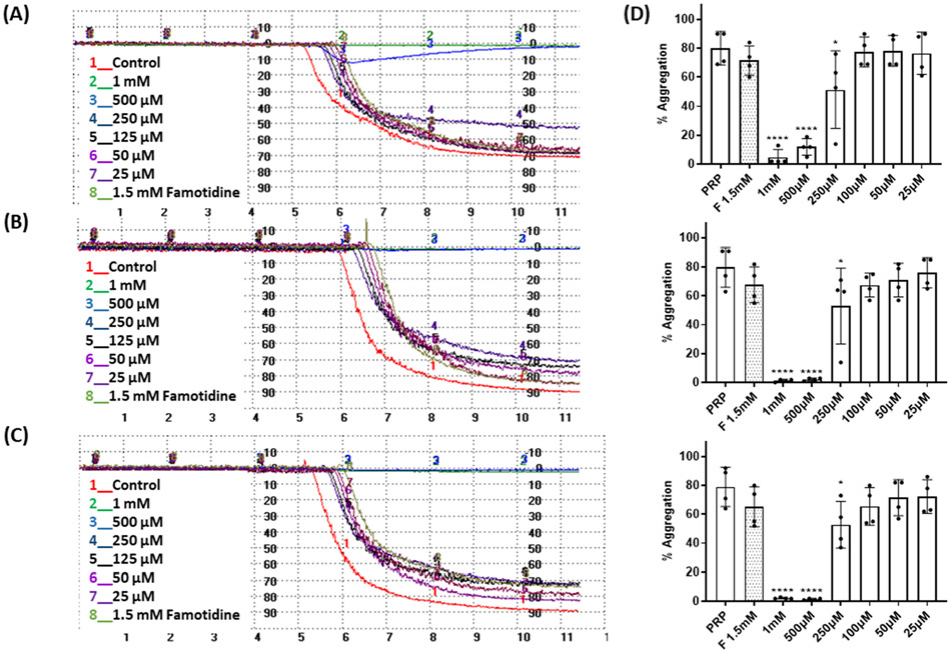
Effect of compounds 1, 2 and 5 on platelet aggregation induced by ADP (10 μM). Representative traces from light transmission aggregometry showing the effect of 1.5 mM famotidine and (A) 1, (B) 2 and (C) 5 on ADP-induced platelet aggregation at various concentrations (25 μM to 1 mM). Untreated platelet rich plasma (PRP) stimulated by ADP was used as the control for platelet aggregation. (D) Quantitative data as per (A)–(C) above. Data are presented as mean ± SD; *n* = 4. One way ANOVA and Dunnett's multiple comparison test; ****P < 0.0001 *vs.* control (PRP in the absence of compounds). Famotidine-F.

The effect of the four compounds, showing good to low affinity for H2 and α2C-ARs (compounds 1–4), on platelet aggregation was explored. Additionally, to elucidate the impact of IMHBs on platelet function, compounds 5 and 6 were also studied. Pre-incubation of platelets with 1, 2 and 5 significantly inhibited collagen- and ADP-induced platelet aggregation at the highest concentrations tested being 1 mM and 500 μM as shown in Fig. 3 and 4, respectively. However, compounds 3, 4 and 6 did not show any inhibitory effect on collagen-induced platelet aggregation (Fig. S8†) and, accordingly, further experiments were not pursued for those compounds.

**Fig. 5:**
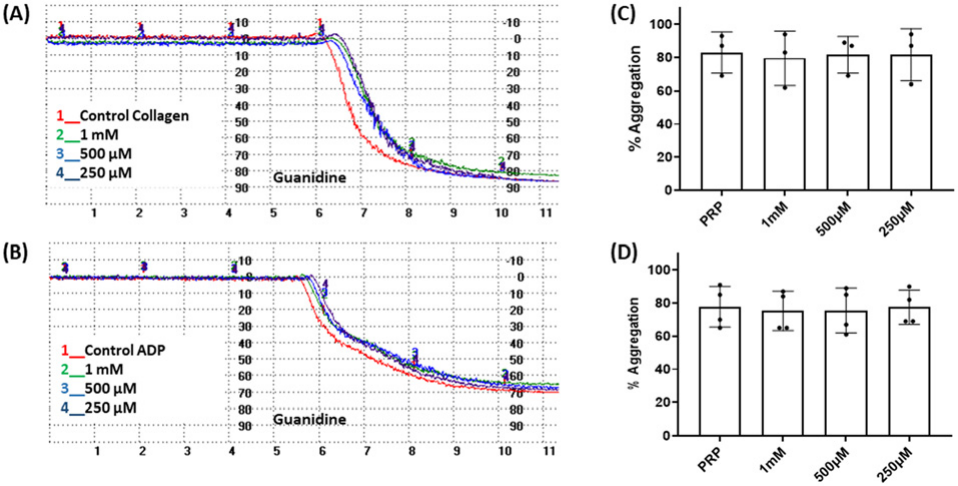
Effect of guanidine on platelet aggregation induced by collagen (2 μg mL^−L^) and ADP (10 μM). Representative traces from light transmission aggregometry demonstrating the absence of the effect of guanidine (250–1.5 mM) on (A) collagen-induced aggregation and (B) ADP-induced aggregation. Quantitative analysis of (C) collagen-induced aggregation, *n* = 3, one way ANOVA *p* = 0.9929 and (D) ADP-induced aggregation, *n* = 4, one way ANOVA *p* = 0.9859. Data are presented as mean ± SD.

**Fig. 6:**
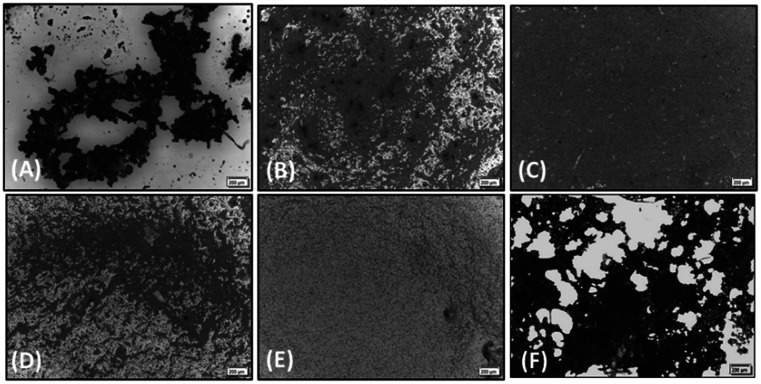
Optical microscopy. Representative images of the effect of (A) guanidine, 1 mM; (B) compound 1, 1 mM; (C) compound 2, 1 mM and (D) compound 5, 1 mM, on platelet aggregation induced by collagen. (E) Resting platelets and (F) collagen (2 μg mL^−L^)-stimulated platelets are shown for comparison. The light grey areas represent non-aggregated platelets and the darker areas show platelet aggregates. Scale bar 200 μm.

Considering that the guanidino functionality was common to the three active compounds, it was deemed necessary to verify whether they may be interacting with plasma proteins through this moiety, leading to a denaturation event thereby preventing the effect of agonist-induced platelet aggregation. A control experiment using guanidine hydrochloride was designed and we found that guanidine did not exert any effect on ADP- or collagen-induced platelet aggregation at any of the concentrations tested (Fig. 5), and the results obtained in these experiments were corroborated by optical microscopy as shown in Fig. 6. Additionally, the three compounds showing inhibition of platelet aggregation are not toxic to platelets at high concentrations (see Fig. S9†), corroborating that the observed antiaggregatory effects are not the result of an increase in the light transmittance in the LTA due to potential platelet's lysis.

### Effect of compounds 1, 2 and 5 on the expression of PAC-1 and P-selectin in platelet activation induced by collagen

**Fig. 7:**
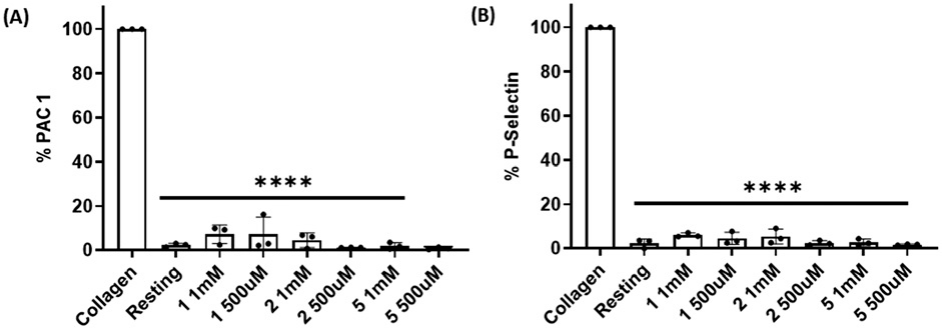
Effect of compounds 1, 2 and 5 on PAC-1 and P-selectin expression in collagen-induced platelet aggregation. Compounds 1, 2 and 5 (1 mM and 500 μM) significantly reduced the expression of both (A) PAC-1 and (B) P-selectin in collagen-activated platelets. Resting platelets and collagen-stimulated platelets in the absence of the compounds were used as controls. Data are presented as mean ± SD; *n* = 3, one way ANOVA and Dunnett's multiple comparison test; ****P < 0.0001 *vs.* control (collagen-stimulated platelets in the absence of compounds).

Platelet expression of both PAC-1 and P-selectin was significantly downregulated when platelets were pre-incubated with compounds 1, 2 and 5 and stimulated with collagen at all concentrations tested when compared with collagen-stimulated platelets in the absence of the compounds (Fig. 7, S10 and S11†).

### Effect of compounds 1, 2 and 5 on tumour cell-induced platelet aggregation (TCIPA)

**Fig. 8:**
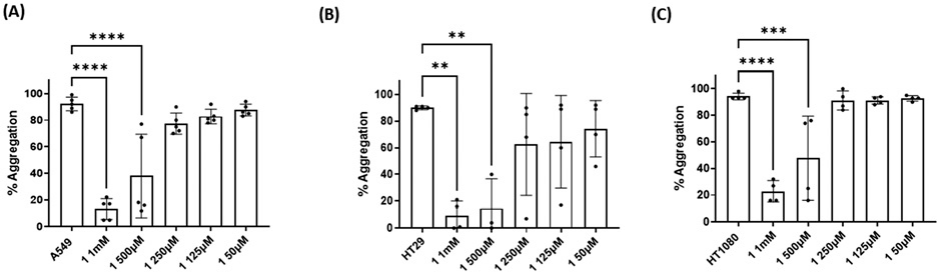
Results of the effect of compound 1 on TCIPA by (A) A549, (B) HT29 and (C) HT1080 cells. One way ANOVA and Dunnett's multiple comparison test; *n* ≥ 4; **p < 0.01; ***p < 0.001 and ****P < 0.0001 *vs.* control.

**Fig. 9:**
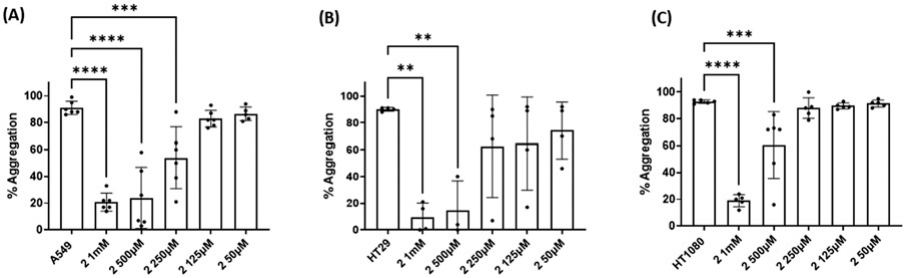
Results of the effect of compound 2 on TCIPA by (A) A549, (B) HT29 and (C) HT1080 cells. One way ANOVA and Dunnett's multiple comparison test; *n* ≥ 4; **p < 0.01; ***p < 0.001 and ****P < 0.0001 *vs.* control.

**Fig. 10:**
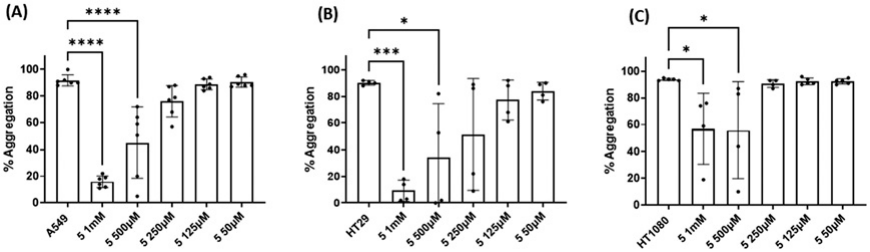
Results of the effect of compound 5 on TCIPA by (A) A549, (B) HT29 and (C) HT1080 cells. One way ANOVA and Dunnett's multiple comparison test; *n* ≥ 4; *p < 0.05; ***p < 0.001 and ****P < 0.0001 *vs.* control.

**Fig. 11:**
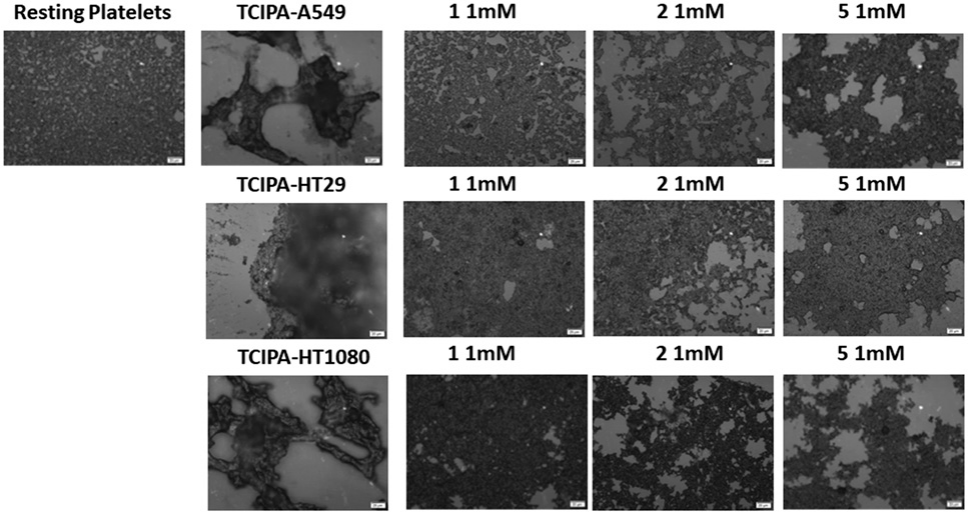
Optical microscopy. Representative images of the effect of compounds 1, 2 and 5 (1 mM) on TCIPA by A549, HT29 and HT1080 cells. Resting platelets and cancer cell-stimulated platelets forming big aggregates are shown for comparison. Scale bar 20 μm.

Three cancer cell lines were used for the TCIPA experiments: A549 (lung carcinoma), HT29 (colon adenocarcinoma) and HT1080 (fibrosarcoma). As shown in Fig. 8–10, TCIPA was significantly inhibited when platelets were preincubated with compounds 1, 2 and 5 at 500 μM and 1 mM. The effect of the compounds on TCIPA was also corroborated by optical microscopy (Fig. 11).

## Discussion

The involvement of the H2 histamine and the α2-AR receptors on platelet aggregation has been previously described. It has been reported that four H2 receptor antagonists (*i.e.* cimetidine, ranitidine, famotidine and roxatidine) have anti-aggregatory activity^27,44^ and that the use of antidepressants or antipsychotic drugs is associated with impaired platelet function.^39,40^ We found that compounds 1 and 2, with high affinity towards H2 receptors and low affinity towards α2(A,C)-AR, as well as their structural analogue compound 5, known to be an α2-AR ligand, were able to inhibit ADP- and collagen-induced platelet aggregation and these results were corroborated by optical microscopy. Our findings correlate well with the mentioned previous studies that showed that different H2 receptor antagonists were able to inhibit platelet aggregation induced by various agonists.^27^ In addition, our results indicate that the observed antiplatelet activity of these three compounds is most likely the result of a direct interaction with receptors in the platelets as guanidine hydrochloride (functionality common to the three derivatives), known to denature collagen and other plasma proteins, had no impact on ADP- and collagen-induced platelet aggregation. Altogether, these results indicate that compounds 1, 2 and 5 display strong antiplatelet activity, at the concentration tested, regardless of the agonist used to induce platelet aggregation.

Since the actual target responsible for the antiplatelet activity of these compounds is not totally known and the full binding screening for compounds 5 and 6 could not be carried out, it is difficult to establish structure–activity relationships. However, in general, it can be concluded that considering the affinity constants provided by the PSDP for compounds 1–4, the lack of antiplatelet activity of compounds 3 and 4 is understandable, since they do not show any binding to α2(A,C)-AR and/or H2 histamine receptors (see Table 1).

Considering the comparable results on platelet aggregation obtained for 1 and 2, which exhibit IMHBs, and for 5, which cannot form IMHBs, it seems complicated to attribute the activity observed to the formation of the mentioned IMHBs. Furthermore, 5 exhibits the most potent antiplatelet effect, even at 250 μM, despite being an α2-AR agonist as previously reported by us.^30^ These results indicate that the antagonism of platelet α2-AR may not be solely responsible for the antiaggregatory effects exhibited by this series of compounds and that alternative targets may also be involved.

Studies have shown that the interaction of AD with α2-AR potentiates human platelet activation and that the resulting platelet adhesion, secretion and aggregation can be inhibited by nicergoline, which is an antagonist of α1A-ARs, α2-ARs and 5-HT1A serotonin receptors.^45^ Additionally, the clonidine analogue UK-14304, which is a potent α2-AR agonist, is almost as effective as AD potentiating human platelet aggregation.^46^ Interestingly, it has been found that clonidine, an α2-AR agonist with an imidazoline moiety in its structure, exerts different effects on platelet aggregation than other α2-AR agonists that do not contain such an imidazoline moiety. Thus, a study of the effect of several imidazoline-carrying derivatives showed that a series of imidazoline derivatives studied (*i.e.* efaroxan, idazoxan, tolazoline or clonidine) effectively inhibit NA induced platelet aggregation.^47^ Therefore, the receptor profiles for the α2-AR antagonists 1 and 2, which showed that these ligands exhibit affinity for the α2C-AR subtype whilst 2 also binds to the α2A-AR, and the fact that compounds 2 and 5 are imidazoline derivatives could shed some light on the antiplatelet activity observed. Even more relevant is the fact that platelets possess serotonin 5-HT2A receptors^48–50^ and that two polymorphisms in the 5-HT2A receptor gene have been suggested to be associated with serotonin-induced platelet aggregation.^51^ Remarkably, compounds 1 and 2 have a high/medium affinity for 5-HT2(A,B,C) serotonin receptors (see Table 1).

Next, we studied the expression of platelet receptors in stimulated platelets in the presence/absence of compounds 1, 2 and 5. The two most predominant and widely used platelet activation markers are P-selectin and activated GPIIb/IIIa. We found that all three compounds at concentrations of 1 mM and 500 μM caused significant inhibition in PAC-1 and P-selectin expression when compared with stimulated platelets in the absence of compounds. These data further support that these compounds can inhibit platelet aggregation without exhibiting platelet toxicity.

At present, it is unclear whether antagonism at H2 and α2-ARs or at other platelet cell surface receptors may contribute to the antiplatelet activity of these three ligands. Therefore, and in order to unravel their exact mechanism of action, a thorough mechanistic investigation would be desired to establish the pathways involved in their antiplatelet effect. The experiments on platelet function were conducted *ex vivo* on platelet rich plasma using a light transmission aggregometer. This method is widely used to study platelet function under ‘quasi’ static conditions and does not take into account the *in vivo* pharmacodynamics of antiplatelet drugs. Therefore, further experiments using full blood systems under flow conditions (for example T-TAS)^52–54^ and/or *in vivo* will be the next steps in our investigation.

Finally, we investigated the effect of 1, 2 and 5 on TCIPA. Platelets can participate in cancer progression. Indeed, tumour cells have been shown to strategically use TCIPA to influence the hematogenous metastasis of tumour cells and to promote tumour cell growth.^8–11,55–57^ In addition, there is strong evidence that cancer cells have the ability to aggregate platelets through surface receptors and signalling molecules, or direct contact.^58^ Three different cell lines were used to study the potential inhibitory effects of novel compounds in TCIPA, HT29 (colon adenocarcinoma), HT1080 (fibrosarcoma) and A549 (lung carcinoma) cells. We found that the compounds tested were capable of modulating TCIPA as shown by LTA and optical microscopy.

## Experimental

### Compounds

Compounds 1 to 6 were synthesised as reported previously by our group.^30,32,33^

### PDSP screening methods

The screening procedure involves a two-step process in which all the compounds are screened in the primary assay to determine whether they displayed inhibition of binding (expressed as mean% − inhibition, *n* = 4 determinations) of a known radioligand at each receptor subtype. The default concentration for the primary assay is 10 μM with significant inhibition considered to be >50%.^59^ Compounds exhibiting >50% inhibition for a given receptor subtype in the primary assay progress to the secondary assay. This allows for a distinct affinity value (*K*_i_) to be measured from radioligand competition binding isotherms derived from competition binding assays using the same known radioligand as in the primary assay and a reference ligand for comparison.^36^

### Blood collection and platelet preparation

Approval for this study was obtained from the School of Pharmacy and Pharmaceutical Sciences Research Ethics Committee (2015-06-01). Following informed consent, blood was withdrawn from healthy volunteers who had not taken any medication known to interfere with platelet function for at least two weeks prior to the study. Platelet isolation was carried out as previously described.^60^ Briefly, platelet rich plasma (PRP) was obtained by blood centrifugation at 250 × *g* for 20 minutes at room temperature. Washed platelets (WPs) were prepared by centrifugation of prostacyclin treated PRP at 900 × *g* for 10 minutes at room temperature and resuspended in Tyrode's salt solution (Sigma, Ireland). Platelet poor plasma (PPP) was prepared by centrifugation of PRP at 13 000 rpm for 10 minutes at room temperature. The platelet count was adjusted to 250 000 platelets per μL before experiments using a Beckman Coulter Z1 series Coulter Counter (Labplan, Ireland).

### Cell culture

Human cancer cell lines, HT29 (colon adenocarcinoma), HT1080 (fibrosarcoma) and A549 (lung carcinoma), were obtained from the European Collection of Authenticated Cell Cultures (ECACC). All three cell lines were cultured at 37 °C in a humidified atmosphere of 5% CO_2_. For carrying out TCIPA experiments, cells were detached using 7 mM EDTA in Dulbecco's phosphate buffered saline (DPBS) (Sigma, Ireland) and centrifuged at 300 × *g* for 5 minutes. Cells were then washed and resuspended in Tyrode's salt solution. The Beckman Coulter Z1 series Coulter Counter (Labplan, Ireland) was used to determine cell concentrations. Samples were further diluted to the final concentrations required to run aggregation experiments using light transmission aggregometry.

### Light transmission aggregometry (LTA)

Platelet aggregation was studied using an eight-channel Platelet Aggregation Profiler Model PAP-8E (Biodata Corporation, Ireland). The percentage of platelet aggregation was calculated by the PAP-8E software where the amount of light passing through a cuvette with the blank (PPP or Tyrode's salt solution) was considered as 100% aggregation. To investigate if the novel H2 receptor antagonist could exert any inhibitory effect in platelet function, platelets were incubated for five minutes in the presence or absence of the compounds at various concentrations and then platelet aggregation was induced by collagen (2 μg mL^−L^) or ADP (10 μM) and their effect was monitored using the software until a plateau was reached. Famotidine at 1.5 mM was used as an anti-H2 internal control.

For TCIPA experiments, and in order to determine the concentration of cells to be used for further experiments, a wide range of A549, HT1080 and HT29 cell concentrations (1000; 5000; 10 000; 20 000; and 50 000 cells per mL) were added to a constant concentration of platelets (250 000 platelets per μL), and the lag phase (the time that takes for platelets to aggregate after adding the cancer cells) and the percentage of aggregation were measured. It was found that 1000 A549 cells per mL, 10 000 HT29 cells per mL and 20 000 HT1080 cells per mL induced maximal platelet aggregation, and those concentrations were therefore chosen for pharmacological modulation of TCIPA. For this purpose, WPs were incubated with the compounds for 5 minutes before TCIPA was initiated by the addition of cancer cells and the platelet aggregation was monitored for up to 40 minutes.

### Cytotoxicity assay

CytoTox-ONE™ Homogeneous Membrane Integrity Assay (Promega, USA) was used to study the potential cytotoxicity effect of the different compounds on platelets following the manufacturer recommendations. Compounds 1, 2 and 5 were incubated with WPs at 500 μM and 1 mM for 10 minutes. Platelet samples from the same donors were lysed and taken as 100% cytotoxicity for comparison. The effect of the vehicle (DMSO) was also measured, and a sample of untreated WPs was used as a control.

### Optical microscopy

Samples from LTA experiments were fixed using 2% paraformaldehyde solution for 30 minutes at 37 °C. Afterwards, samples were mounted on slides using a Cytospin™ 4 Cytocentrifuge (Fisher Scientific, Ireland) and observed under a BX51M Olympus Microscope (Mason Technology, Ireland), and photomicrographs were captured using a digital camera.

### Flow cytometry

Platelet expression of activated GPIIb/IIIa and P-selectin induced by collagen in the presence and absence of the compounds was examined by flow cytometry. Once platelets for the control (in the absence of compounds and stimulated with collagen) reached 50% maximal light transmission, samples were taken and incubated with the monoclonal FITC conjugated mouse anti-human PAC-1 antibody (BD Biosciences, Ireland) against GPIIb/IIIa and with PE conjugated mouse anti human CD62P antibody (P-selectin) (BD Biosciences, Ireland) in the dark for 5 minutes. After this time, samples were diluted and analysed using a BD ACCURI C6 (Biosciences, UK). Resting platelets and platelets activated with collagen were used as negative and positive controls, respectively.

### Statistical analysis

Data were obtained from experiments carried out with at least three different blood donors and analysed using GraphPad Prism 9.5.1 (GraphPad Software, La Jolla, CA, USA). The results are expressed as mean ± SD. Statistical analysis was performed using one-way ANOVA and Dunnett's multiple comparison test. Differences between groups were considered statistically significant when *P* < 0.05.

## Conclusions

Platelets play an important role in both physiological and pathological conditions, including cardiovascular diseases and cancer. It has been previously shown that histamine and adrenaline may enhance platelet aggregation and that histamine H2 or α2C-AR antagonists may, therefore, inhibit platelet aggregation.^28,44^ In this work, several compounds from Rozas' in-house library were screened for their affinity towards a number of neurological targets including monoamine transporters (DAT, NET, SERT), and dopaminergic (D1-like: D1 and D5, and D2-like: D2 and D4), serotonergic (5-HT1A, 5-HT1B, 5-HT1D, 5-HT1E, 5-HT2A, 5-HT2B, 5-HT2C, 5-HT3), sigma (σ1 and σ2), adrenergic (α1A-, α2A-, α2C-, and β2-ARs) and histamine (H2 and H3) receptors. From this screening, four compounds (1–4) were identified with good adrenergic (α2C) and histaminergic (H2) affinity (see Table 1). Based on the structure of the pyridine-2-yl guanidines with the best affinity towards H2 receptors (compounds 1 and 2), which showed IMHBs between the pyridine N atom and the guanidinium group, other two analogues were selected from the ‘in-house’ library (compounds 5 and 6). These two compounds could not form IMHBs and had previously shown to be good α2-AR ligands with nM *K*_i_ values.^31,33^ The six compounds selected were then tested to determine their effect on platelet aggregation, and we found that three of these compounds (1, 2 and 5) were able to inhibit platelet aggregation induced by the two platelet agonists used, collagen and ADP. In addition, these three compounds were tested on TCIPA showing that they were also able to modulate TCIPA using different cell lines. In conclusion, this study provides pioneering evidence of the inhibitory effect of three guanidine/imidazoline aryl derivatives with affinity towards H2 histamine and α2(A,C)-AR receptors as well as 5-HT2(B,C) serotonin receptors on platelet function. In addition, these compounds were also able to modulate TCIPA. Further studies are guaranteed to study the effect of these compounds *in vivo* and elucidate their safety profile.

## Data availability

The data supporting this article have been included as part of the ESI.†

## Author contributions

N. K. H. and A. P. K. contributed equally to this research. Conceptualization, I. R., C. M. and M. J. S. M.; methodology, I. R., C. M. and M. J. S. M.; data analysis, N. K. H., A. P. K., Y. H., I. R., C. M. and M. J. S. M.; experiments, N. K. H., A. P. K., Y. H., T. K. and H. B. L.; writing and editing, E. W., I. R., C. M. and M. J. S. M.; supervision, I. R., C. M. and M. J. S. M. All authors have read and agreed to the published version of the manuscript.

## Conflicts of interest

There are no conflicts to declare.

## Supplementary Material

MD-OLF-D4MD00793J-s001
