# Supplementary material for: Effect of mono-guanidine-like derivatives on platelet aggregation and tumour cell induced platelet aggregation
Source: RSC Med Chem. 2025 Jan 31;16(4):1695–704. doi: 10.1039/d4md00793j (PMC11865921; doi:10.1039/d4md00793j)
Supplement: MD-016-D4MD00793J-s001 [file MD-016-D4MD00793J-s001.pdf]

Supplementary Information (SI) for RSC Medicinal Chemistry.
This journal is © The Royal Society of Chemistry 2025

Supplementary Material

Effect of mono-Guanidine-like Derivatives on Platelet Aggregation and Tumour Cell Induced Platelet Aggregation

Nadhim Kamil Hante,[1,2#] Aaron P. Keogh,[3#], Yanni Huang[1], Tanya Kapoor[1], Harriet Bennett-Lenane[1], Eleanor Walsh[1], Isabel Rozas,[3]* Carlos Medina, [1]* Maria Jose Santos-Martinez [1,4]*

[1]*School of Pharmacy & Pharmaceutical Sciences, Trinity College Dublin, The University of Dublin,* Panoz Institute, D02PN40*Dublin (Ireland)*

[2]*Faculty of Pharmacy, University of Kufa, Al-Najaf, Iraq.*

[3]*School of Chemistry, Trinity Biomedical Sciences Institute, Trinity College Dublin, The University of Dublin, 152-160 Pearse Street, D02 R590, Dublin (Ireland)*

[4] *School of Medicine, Trinity Biomedical Sciences Institute, Trinity College Dublin, The University of Dublin, 152-160 Pearse Street, D02 R590, Dublin (Ireland)*

*Corresponding authors: rozasi@tcd.ie, martinc2@tcd.ie, santosmm@tcd.ie

[#]Contributed equally to this research

1. Synthesis details

1.1. General information

All commercial chemicals used were supplied by Sigma Aldrich, Fluorochem, and Santa Cruz Biotechnology and used without further purification unless otherwise stated. Deuterated solvents for NMR were purchased from Apollo or VWR. Solvents for synthesis purposes were used at GPR grade. Anhydrous CH_2Cl_2, THF, CH_3CN and Et_2O were purified with the PureSolv MD-4EN Solvent Purification System. Silica gel 60 (Merck, 230-400 mesh) was used for flash column chromatography. All compounds were subject to purification using silica gel, unless otherwise stated. Analytical thin layer chromatography was carried out with silica gel 60 (fluorescence indicator F254; Merck) and visualised by UV irradiation.

Melting points are uncorrected and were measured with a Stuart SP-10 melting point apparatus. NMR spectra were recorded using Bruker DPX 400 (400.13 MHz for ^{1}H NMR and 100.61 MHz for ^{13}C NMR), Bruker AV 600 (600.13 MHz for ^{1}H NMR and 150.90 MHz for ^{13}C NMR), Bruker AV 400 (400.13 MHz for ^{1}H NMR and 100.61 MHz for ^{13}C NMR) or Agilent MR400 (400.13 MHz for ^{1}H NMR and 100.61 MHz for ^{13}C NMR) instruments. Chemical shifts, δ, are in ppm units downfield from an internal reference. NMR data was processed using MestReNova software. The assignment of the signals was confirmed by 2D spectra (COSY, HMBC, HSQC). MALDI Tof spectra were acquired using a Waters MALDI Q-Tof Premier in positive or negative mode with DCTB (trans-2-[3-(4-tert-butylphenyl)-2-methyl-2-propenylidene] malononitrile) as the MALDI matrix. ESI mass spectra were acquired in positive and negative modes as required, using a Micromass time of flight mass spectrometer (TOF), interfaced to a Waters 2690 HPLC or a Bruker microTOF-Q III spectrometer interfaced to a Dionex UltiMate 3000 LC. APCI experiments were carried out on a Bruker micrOTOF-QIII spectrometer interfaced to a Dionex UltiMate 3000 LC or direct insertion probe in positive or negative modes. Infrared spectra (IR) spectra were recorded on a Perkin-Elmer Spectrum 100 FT-IR spectrometer, equipped with a Universal ATR sampling accessory.

Purity of final hydrochloride salts was determined using HPLC. Purity was assessed using reverse phase HPLC with a diode-array detector scanning wavelengths from 200 to 950 nm. HLPC analysis was carried out using a Varian ProStar system equipped with a Varian Prostar 335 diode array detector and a manual injector (20 µL). For purity assessment, UV detection was performed at 245 nm and peak purity was confirmed using a purity channel. The stationary phase consisted of an ACE 5 C18-AR column (150 mm × 4.6 mm), and the mobile phase used the following gradient system, eluting at 1 mL/min: aqueous formate buffer (30 mM, pH 3.0) for 10 min, linear ramp to 85% methanol buffered with the same system over 25 min, hold at 85% buffered methanol for 10 min. Minimum requirement for purity was set at 95.0%.

1.2. Methods

Method A: *General procedure for the synthesis of Boc-protected guanidine derivatives.*

Each of the corresponding amines (1.0 eq.) in the CH2Cl2 at 0 °C was treated with HgCl2 (1.1 eq.), *N,N'*-di(*tert*-butoxycarbonyl)-thiourea (1.0 eq.) or 1,3-bis(*tert*-butoxycarbonyl)-2-methyl-2-thiopseudourea

(1.0 eq.) and NEt3 (3.1 eq.). The resulting mixture was stirred at 0 °C for 1 h and for the appropriate duration at room temperature until reaction was adjudged complete by TLC analysis. Then the reaction mixture was diluted with EtOAc and filtered through a pad of Celite to remove mercury by-products. The filter cake was rinsed with EtOAc. The organic phase was extracted with water (2 x 30 mL), washed with brine (1 x 30 mL), dried over anhydrous Na2SO4, and concentrated under reduced pressure to give a residue that was purified by silica gel column chromatography, eluting with the appropriate solvent system.

Method B: *General procedure for the synthesis of the Boc-protected 2-iminoimidazolidine derivatives.*

Each of the corresponding amines (1.0 eq.) was treated in the CH2Cl2 at 0 °C with HgCl2 (1.1 eq,), *N,N'*-di(*tert*-butoxycarbonyl)-imidazolidine-2-thione (1.0 eq.) and NEt3 (3.1 eq.). The resulting mixture was stirred at 0 °C for 1 h and for the appropriate duration at room temperature until reaction was adjudged complete by TLC analysis. Then the reaction mixture was diluted with EtOAc and filtered through a pad of Celite to remove mercury by-products. The filter cake was rinsed with EtOAc. The organic phase was extracted with water (2 x 30 mL), washed with brine (1 x 30 mL), dried over anhydrous Na2SO4, and concentrated under reduced pressure to give a residue that was purified by neutral alumina column flash chromatography, eluting with the appropriate solvent system. The residue obtained after the column was recrystallized from the appropriate solvent when required.

Method C: *Procedure for the synthesis of hydrochloride salts.*

The Boc protected derivative (0.5 mmol) was treated with a solution of 50% TFA in CH2Cl2 (excess, 15 mL). After 3-6 h stirring at room temperature, CH2Cl2 and TFA were removed under reduced pressure to afford the trifluoroacetate salt. This salt was dissolved in H2O (10 mL and 1.0 g of activated Amberlite IRA-400 chloride form (excess) was added, and the reaction was stirred overnight. After 24 h the Amberlite was removed by filtration, and the filtrate was washed with CH2Cl2 (2 x15 mL). The aqueous phase was concentrated to dryness under vacuum to yield the hydrochloride salt, 19F NMR confirmed the absence of TFA.

1.3. Synthesis of Compound 1

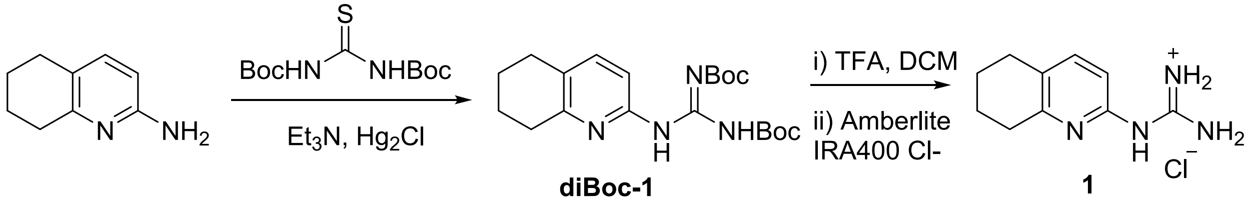

1-(5,6,7,8-Tetrahydroquinolin-2-yl)-2,3-di(*tert*-butoxycarbonyl)guanidine (diBoc-1)[1]

Following method A, a solution of *N,N'*-di(*tert*-butoxycarbonyl)-thiourea (0.10 g, 0.67 mmol) in CH2Cl2 (4 mL) at 0 °C was treated with HgCl2 (0.19 g, 0.71 mmol), 5,6,7,8-tetrahydroquinolin-2-amine (0.21 g, 0.71 mmol) and NEt3 (0.3 mL, 2.36 mmol). The resulting mixture was stirred at 0 °C for 1 h and 18 h at room temperature. The standard work up followed by silica gel column chromatography afforded **diBoc-1** as a white solid. **Yield:** 80% (0.21 g, 0.54 mmol). **M.P.:** 164-166 ºC (Lit. 164-165 ºC).[1] **δH** (400 MHz,

CDCl3): 1.51 (s, 18H, (CH3)3 -1'), 1.75 (m, 2H, CH2-3), 1.83 (m, 2H, CH2-2), 2.69 (t, 2H, *J* 6.3 Hz CH2-4), 2.78 (t, 2H, *J* 6.3 Hz, CH2-1), 7.35 (d, 1H, *J* 8.3 Hz, CH-5), 8.04 (bs, 1H, CH-6), 10.63 (bs, 1H, NH-2'), 11.54 (bs, 1H, NH-3').

1-(5,6,7,8-Tetrahydroquinolin-2-yl)-guanidine hydrochloride (1)[1]

Following method C, **diBoc-1** (0.21 g, 0.54 mmol) was treated with a solution of 50% TFA in CH2Cl2 (10 mL) for 3 h stirring at room temperature. Solvents were removed under reduced pressure to afford the trifluoroacetate salt. This salt was dissolved in H2O (20 mL) and treated with Amberlite IRA 400 chloride form (1.0 g) for 24 h. The resin was removed by filtration and the aqueous solution was washed with CH2Cl2 (2 x 10 mL). Evaporation of the water afforded the pure hydrochloride salt **1** as a brown solid. **Yield**: 88% (0.11 g, 0.47 mmol). **M.P.**: 224-226 °C (Lit. 224-228 °C, decomp.).[1] **δH** (600 MHz, D2O): 1.8 (m, 4H, CH2-2, CH2-2), 2.72 (t, 2H, *J* 6.2 Hz, CH2-4), 2.79 (t, 2H, *J* 6.4 Hz, CH2-1), 6.78 (d, 1H, *J* 8.3 Hz, CH-6), 7.54 (d, 1H, *J* 8.3 Hz,CH-5). **HPLC**: 98% (tR = 27.2 min).

1.4. Synthesis of Compound 2

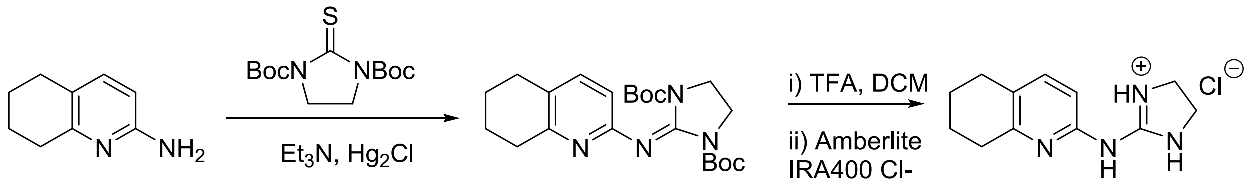

2-(5,6,7,8-Tetrahydro-naphthalen-2-ylimino)- 1,3- di(*tert*-butoxycarbonyl)imidazolidine-guanidine (diBoc-2).[2]

Following method B, a solution of *N,N'*-di(*tert*-butoxycarbonyl)-imidazolidine-2-thione (0.53 g, 3.62 mmol) in CH2Cl2 (5 mL) at 0 °C was treated with HgCl2 (1.08 g, 3.98 mmol), 5,6,7,8-tetrahydroquinolin-2-amine (1.09 g, 3.62 mmol) and NEt3 (1.56 mL, 11.22 mmol). The resulting mixture was stirred at 0 °C for 1 h and 18 h at room temperature. The usual workup followed by neutral alumina column flash chromatography afforded **diBoc-2** as a white solid. **Yield**: 73% (1.1 g, 2.64 mmol). **M.P**: 140-142 (Lit. 140-142 °C).[2] **δH** (400 MHz, CDCl3): 1.28 (s, 18H, (CH3)3-2'), 1.72 (m, 4H, CH2-3 and -4), 2.64 (m, 4H, CH2-2 and -5), 3.77 (s, 4H, CH2-1'), 6.65 (s, 1H, CH-1), 6.69 (d, 1H, *J* 8.0 Hz, CH-6), 6.87 (d,1H, *J* 8.0 Hz, CH-7).

1-(5,6,7,8-Tetrahydroquinolin-2-yl)-2-iminoimidazolidine hydrochloride (2).[2]

Following method C, **diBoc-2** (0.20 g, 0.48 mmol) was treated with a solution of 50% TFA in CH2Cl2 (10 mL) for 3 h stirring at room temperature. Solvents were removed under reduced pressure to afford the trifluoroacetate salt. This salt was dissolved in H2O (20 mL) and treated with Amberlite IRA 400 chloride form (1.0 g) for 24 h. The resin was removed by filtration and the aqueous solution was washed with CH2Cl2 (2 x 10 mL). Evaporation of the water afforded the pure hydrochloride salt **2** as a white solid. **Yield**: 86% (0.10 g, 0.41 mmol). **M.P.**: 208-210 °C (Lit. 208-212 °C).[2] **δH** (600 MHz, DMSO-d6) : 1.74 (m, 2H, CH2-5), 1.81 (m, 2H, CH2-6), 2.70 (t, 2H, *J* 6.2 Hz, CH2-4), 3.02 (t, 2H, *J* 6.2 Hz, CH2-7), 3.73 (s, 4H,

CH2-1), 6.93 (d, 1H, *J* 8.2 Hz, CH-2), 7.55 (d, 1H, *J* 8.2 Hz, CH-3), 8.80 (bs, 2H, NH-2'), 11.89 (bs, 1H, NH-1'). **HPLC**: 96% (tR = 21.7 min).

1.5. Synthesis of Compound 3

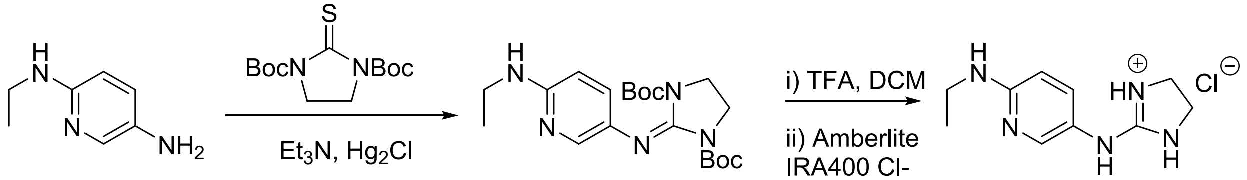

diBoc-3 **3**

1-(6-(Ethylamino)pyridin-3'-yl)-2,3-di(*tert*-butoxycarbonyl)-2-iminoimidazolidine (diBoc-3).[3]

Following method B, a solution of *N,N'*-di(*tert*-butoxycarbonyl)-imidazolidine-2-thione (0.2 g, 1.46 mmol) in CH2Cl2 (5 mL) at 0 °C was treated with HgCl2 (0.44 g, 1.6 mmol), 2-aminoethyl-5-aminopyridine (0.44 g, 1.46 mmol) and NEt3 (0.92 mL, 6.61 mmol). The resulting mixture was stirred at 0 °C for 1 h and 18 h at room temperature. The usual workup followed by neutral alumina column flash chromatography afforded **diBoc-3** as a white solid. **Yield**: 60% (0.35 g, 0.87 mmol). **M.P**: 120-122 °C (Lit 121-123 °C).[3] **δH** (400 MHz, CDCl3): 0.99 (t, 3H, *J* 7.0 Hz, CH3-7), 1.14 (s, 18H, (CH3)3-2), 3.06 (q, 2H, *J* 7.0 Hz, CH2-6), 3.60 (s, 4H, CH2-1), 4.48 (bs, 1H, NH), 6.13 (d, 1H, *J* 8.8 Hz, CH-5), 6.98 (dd, 1H, *J* 8.8 Hz, 2.6 Hz, CH-4), 7.65 (d, 1H, *J* 2.6 Hz, CH-3).

1-(6-(Ethylamino)pyridin-3-yl)-2-iminoimidazolidine dihydrochloride (3).[3]

Following method C, **diBoc-3** (0.35 g, 0.87 mmol) was treated with a solution of 50% TFA in CH2Cl2 (20 mL) for 3 h stirring at room temperature. Solvents were removed under reduced pressure to afford the trifluoroacetate salt. This salt was dissolved in H2O (20 mL) and treated with Amberlite IRA 400 chloride form (1.0 g) for 24 h. The resin was removed by filtration and the aqueous solution was washed with CH2Cl2 (2 x 10 mL). Evaporation of the water afforded the pure hydrochloride salt **3** as a white solid. **Yield**: 90% (0.22 g, 0.78 mmol). **M.P**: Decomp. >210 °C (Lit. >210 °C).[3] **δH** (600 MHz, DMSO-d6): 1.24 (t, 3H, *J* 7.1 Hz, CH3-1), 3.47 (q, 2H, *J* 7.1 Hz, CH2-2), 3.65 (s, 4H, CH2-6), 7.18 (d, 1H, *J* 9.4 Hz, CH-3), 7.78 (d, 1H, *J* 9.4 Hz, CH-4), 7.92 (s, 1H, CH- 5), 8.58 (bs, 2H, NH-2'), 10.65 (bs, 2H, NH-1). **HPLC**: 96% (tR = 3.2 min).

1.6. Synthesis of Compound 4

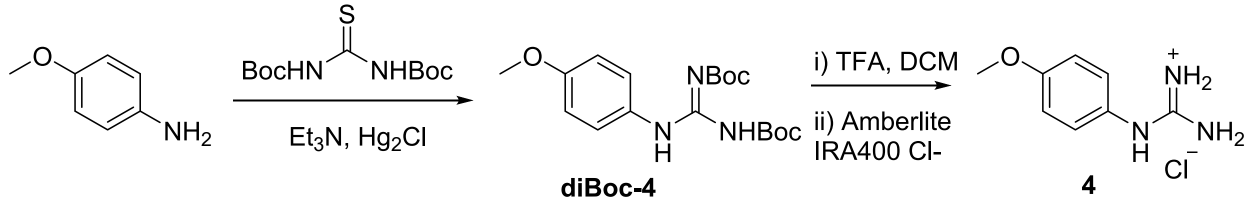

diBoc-4 **4**

1-[2,3-Di(*tert*-butoxycarbonyl)guanidino]-4-methoxybenzene (diBoc-4).[4]

Following method A, a solution of *N,N'*-di(*tert*-butoxycarbonyl)-thiourea (0.37 g, 3.0 mmol) in CH2Cl2 (5 mL) at 0 °C was treated with HgCl2 (0.89 g, 3.3 mmol), 4-methoxyaniline (0.83 g, 3.0 mmol) and NEt3 (1.3 mL, 9.3 mmol). The resulting mixture was stirred at 0 C for 1 h and 18 h at room temperature. The usual workup followed by silica gel column chromatography afforded **diBoc-4** as a white solid. **Yield**: 80% (0.88 g, 2.4 mmol). **M.P**: 180-182 °C (Lit. 181-183 °C).[4] **δH** (400 MHz, CDCl3): 1.52 (s, 9H, (CH3)3-1), 1.56 (s, 9H, (CH3)3-2), 3.85 (s, 3H, CH3-5), 6.90 (d, 2H, *J* 8.0 Hz, CH-4), 7.50 (d, 2H, *J* 8.0 Hz, CH-3), 10.18 (bs, 1 H, NH-1'), 11.70 (bs, 1 H, NH-2').

1-Guanidino-4-methoxybenzene hydrochloride (4).[4]

Following method C, **diBoc-4** (0.37 g, 1.0 mmol) was treated with a solution of 50% TFA in CH2Cl2 (20 mL) for 3 h stirring at room temperature. Solvents were removed under reduced pressure to afford the trifluoroacetate salt. This salt was dissolved in H2O (20 mL) and treated with Amberlite IRA 400 chloride form (1.0 g) for 24 h. The resin was removed by filtration and the aqueous solution was washed with CH2Cl2 (2 x 10 mL). Evaporation of the water afforded the pure hydrochloride salt **4** as a white solid. **Yield**: 94% (0.19 g, 0.94 mmol). **M.P**: 138-142 °C (Lit. 139-141 °C).[4] **δH** (400 MHz, D2O): 3.87 (s, 3H, CH3-1), 7.08 (d, 2H, *J* 8.8 Hz, CH-2), 7.30 (d, 2H, *J* 8.8 Hz, CH-3). **HPLC**: 99% (tR = 17.56 min).

1.7. Synthesis of Compound 5

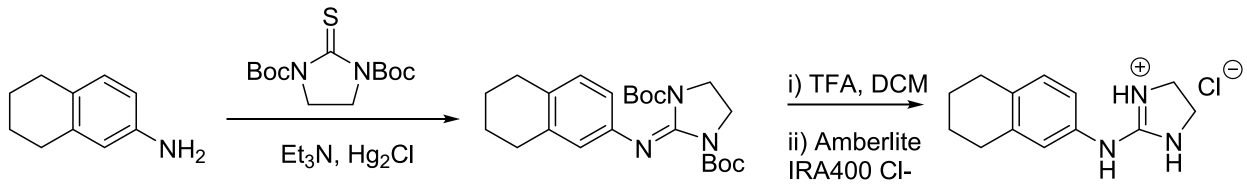

diBoc-5 **5**

2-(5,6,7,8-tetrahydro-naphthalen-2-ylimino)-imidazolidine-1,3-dicarboxylic acid di-*tert*-butyl ester (diBoc-5).[2]

Following method B, a solution of *N,N'*-di(*tert*-butoxycarbonyl)-imidazolidine-2-thione (0.53 g, 3.62 mmol) in CH2Cl2 (5 mL) at 0 °C was treated with HgCl2 (1.08 g, 3.98 mmol), 5,6,7,8-tetrahydro naphthalen-2-amine (1.09 g, 3.62 mmol) and NEt3 (1.56 mL, 11.22 mmol). The resulting mixture was stirred at 0 °C for 1 h and 18 h at room temperature. The usual workup followed by neutral alumina column flash chromatography afforded **diBoc-5** as a white solid. **Yield**: 73% (1.1 g, 2.64 mmol). **M.P**: 140-142 (Lit. 140-142 °C).[2] **δH** (400 MHz, CDCl3): 1.28 (s, 18H, (CH3)3-2'), 1.72 (m, 4H, CH2-3 and -4), 2.64 (m, 4H, CH2-2 and -5), 3.77 (s, 4H, CH2-1'), 6.65 (s, 1H, CH-1), 6.69 (d, 1H, *J* 8.0 Hz, CH-6), 6.87 (d, 1H, *J* 8.0 Hz, CH-7).

Imidazolidin-2-ylidene-(5,6,7,8-tetrahydronaphthalen-2-yl)amine hydrochloride (5).[2]

Following method C, **diBoc-5** (0.42 g, 1.0 mmol) was treated with a solution of 50% TFA in CH2Cl2 (20 mL) for 3 h stirring at room temperature. Solvents were removed under reduced pressure to afford the trifluoroacetate salt. This salt was dissolved in H2O (20 mL) and treated with Amberlite IRA 400 chloride form (1.0 g) for 24 h. Then, the resin was removed by filtration and the aqueous solution was washed with CH2Cl2 (2 x 10 mL). Evaporation of the water afforded the pure hydrochloride salt **5** as a white solid. **Yield**: 92% (0.23 g, 0.92 mmol). **M.P**: 88-90°C (Lit. 87-89 °C).[2] **δH** (400 MHz, D2O): 1.77 (m, 4H, CH2-4, CH2-5), 2.76 (m, 4H, CH2-3, CH2-6), 3.75 (s, 4H, CH2-1), 7.01 (m, 2H, CH-2, CH-8), 7.20 (d, 1H, *J* 7.8 Hz, CH-7). **HPLC**: 97.3% (tR = 27.5 min).

1.8. Synthesis of Compound 6

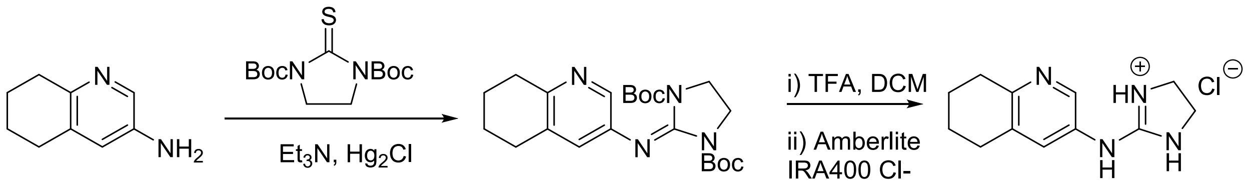

diBoc-6 **6**

2-(5,6,7,8-tetrahydro-quinolin-3-ylimino)-imidazolidine-1,3-dicarboxylic acid di-*tert*-butyl ester (diBoc-6).[3]

Following method B, a solution of *N,N'*-di(*tert*-butoxycarbonyl)-imidazolidine-2-thione (0.53 g, 3.62 mmol) in CH2Cl2 (5 mL) at 0 °C was treated with HgCl2 (1.08 g, 3.98 mmol), 5,6,7,8-tetrahydroquinolin-3-amine (1.09 g, 3.62 mmol) and NEt3 (1.56 mL, 11.22 mmol). The resulting mixture was stirred at 0 °C for 1 h and 18 h at room temperature. The usual workup followed by neutral alumina column flash chromatography afforded **diBoc-6** as a white solid. **Yield**: 81%. **M.P**: 128-131 °C, clean melt.[3] **δH** (400 MHz, CDCl3): 1.21 (s, 18H, CH3, Boc), 1.60-1.66 (m, 2H, CH2), 1.68-1.75 (m, 2H, CH2), 2.57 (app. t, 2H, J 6.0, 6.3, CH2), 2.71 (app. t, 2H, J 6.0, 6.3, CH2), 3.71 (s, 4H, CH2), 6.83 (d, 1H, J 2.4, Ar), 7.94 (d, 1H, J 2.4, Ar).

Imidazolidin-2-ylidene-(5,6,7,8-tetrahydroquinoline-3-yl)amine hydrochloride (6).[3]

Following method C, **diBoc-6** (0.42 g, 1.0 mmol) was treated with a solution of 50% TFA in CH2Cl2 (20 mL) for 3 h stirring at room temperature. Solvents were removed under reduced pressure to afford the trifluoroacetate salt. This salt was dissolved in H2O (20 mL) and treated with Amberlite IRA 400 chloride form (1.0 g) for 24 h. Then, the resin was removed by filtration and the aqueous solution was washed with CH2Cl2 (2 x 10 mL). Evaporation of the water afforded the pure hydrochloride salt **6** as a white solid. **Yield**: 95%. **M.P**: >210 °C.[3] **δH** (400 MHz, D2O): 1.22 (t, 3H, J = 7.1, CH3), 3.45 (q, 2H, J = 7.1, CH2), 3.65 (s, 4H, CH2), 7.15 (d, 1H, J = 9.4, CH2), 7.76 (d, 1H, J = 9.4, Ar), 7.92 (s, 1H, Ar), 8.55 (br s, 2H, NH), 10.63 (br s, 1H, NH). **HPLC**: 97.9% (tR = 3.2 min).

2. PDSP screening procedure

The screening procedure involves a two-step process in which all of the compounds are screened in the *primary* assay to determine whether they displayed inhibition of binding (expressed as mean %-inhibition, N = 4 determinations) of a known radioligand at each receptor subtype. The default concentration for the *primary* assay is 10 µM with significant inhibition considered to be >50%.[5] Compounds exhibiting >50% inhibition for a given receptor subtype in the *primary* assay progress to the *secondary* assay. This allows for a distinct affinity value (K_i) to be measured from radioligand competition binding isotherms derived from competition binding assays using the same known radioligand as in the *primary* assay and a reference ligand for comparison.[6]

2.1. Details of the primary and secondary assays

Dopamine transporter (DAT)

The radioligand used for the primary assay to measure inhibition of the DAT was [3H]WIN3542.

Noradrenaline transporter (NET)

The radioligand used for the primary assay to measure inhibition of the NET was [3H]-nisoxetine.

Serotonin transporter (SERT)

The radioligand used for the primary assay to measure inhibition of the SERT was [3H]-citalopram.

Dopaminergic receptors

The selected compounds were screened for affinity at the following dopaminergic receptors: D1-like (D1 and D5) and D2-like (D2, D3 and D4) receptors. As such, only two different radioligands are required to screen for affinity at all five DA receptors in the primary assay: D1-like are screened using the tritiated D1 antagonist [3H]-SCH23390, whilst D2-like screening utilizes the D2 antagonist [3H]-*N*-methylspiperone. For the secondary assay the antipsychotic chlorpromazine, a potent D2 antagonist, was utilised as the reference ligand for D2[7] [3] and the typical anstipsychotic (+)-butaclamol as the reference ligand for D1.

2.2. Results from secondary assays in PDSP screening

Dopamine receptors

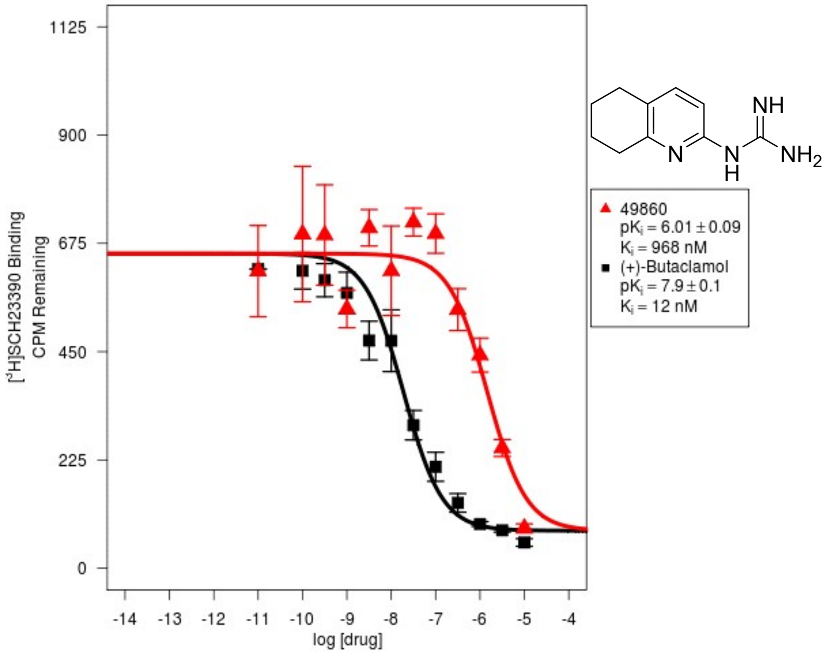

Figure S1. Competition binding isotherms for **1** with the D1 receptor (reference: (+)-Butaclamol; "hot ligand": [^{3}H]-SCH23390).

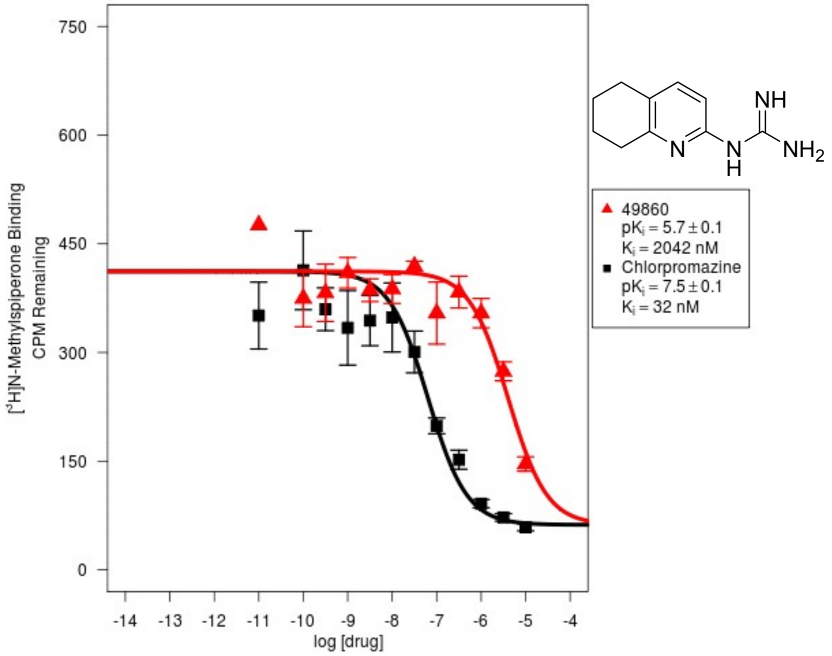

Figure S2. Competition binding isotherm for **1** with the D4 receptor (reference: Chlorpromazine; "hot ligand": [^{3}H]-*N*-methylspiperone).

__Serotonergic (5-HT) Receptors__

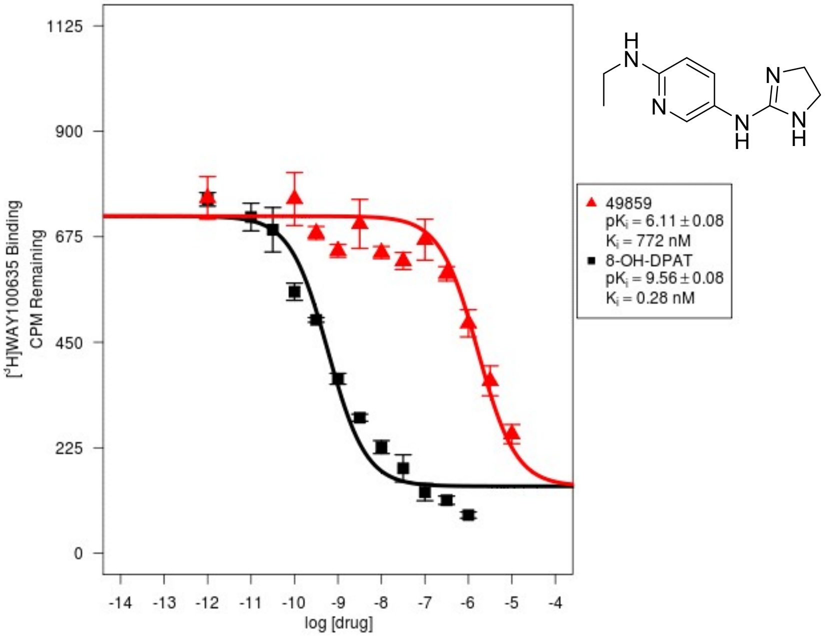

Figure S3. Competition binding isotherm for **3** with the 5-HT1A receptor (reference: 8-OH-DPAT; “hot ligand”: [^{3}H]-WAY100635).

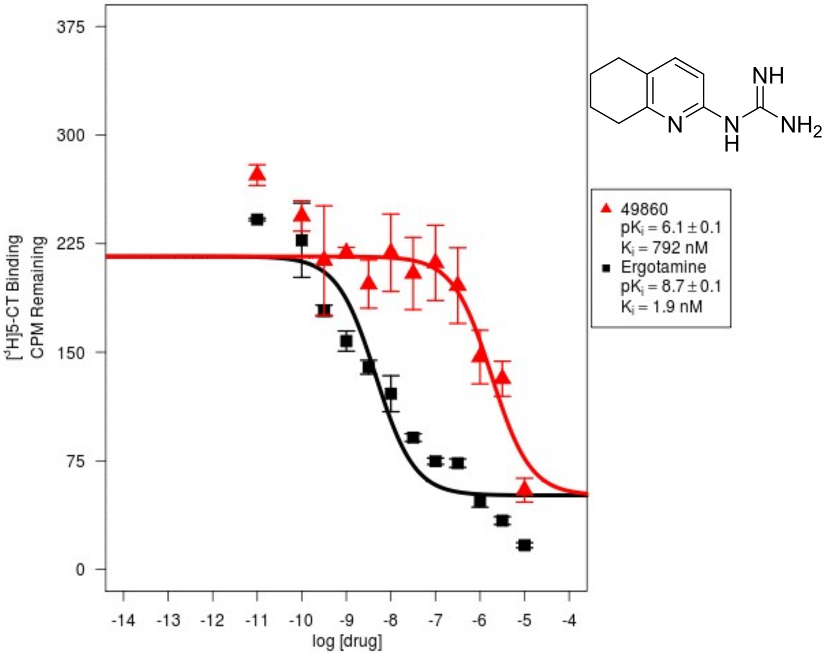

Figure S4. Competition binding isotherm for **1** with the 5-HT$_{1B}$ receptor (reference: Ergotamine; “hot ligand”: [^{3}H]-5-CT).

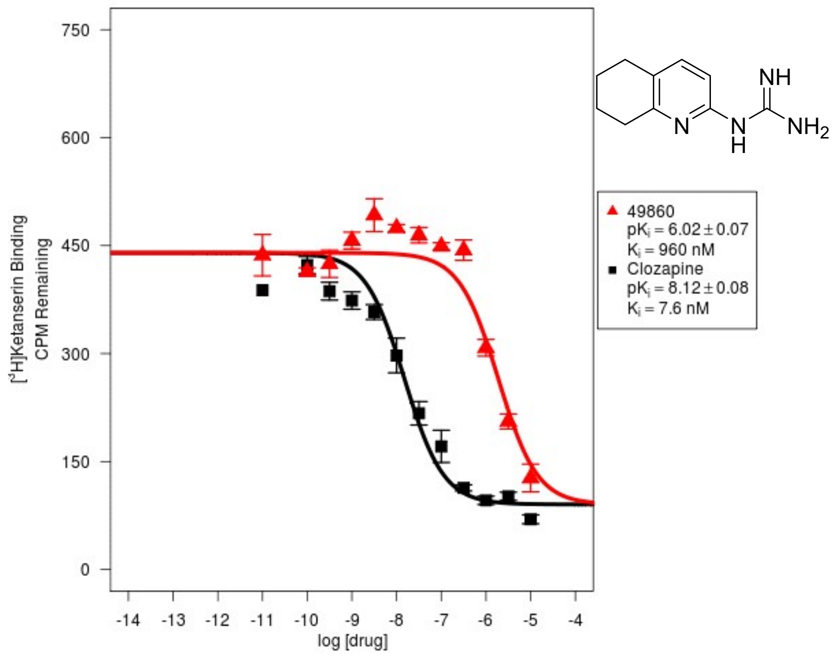

Figure S5. Competition binding isotherm for **1** with the 5-HT_{2A} receptor (reference: Clozapine; "hot ligand": [^{3}H]-ketanserin).

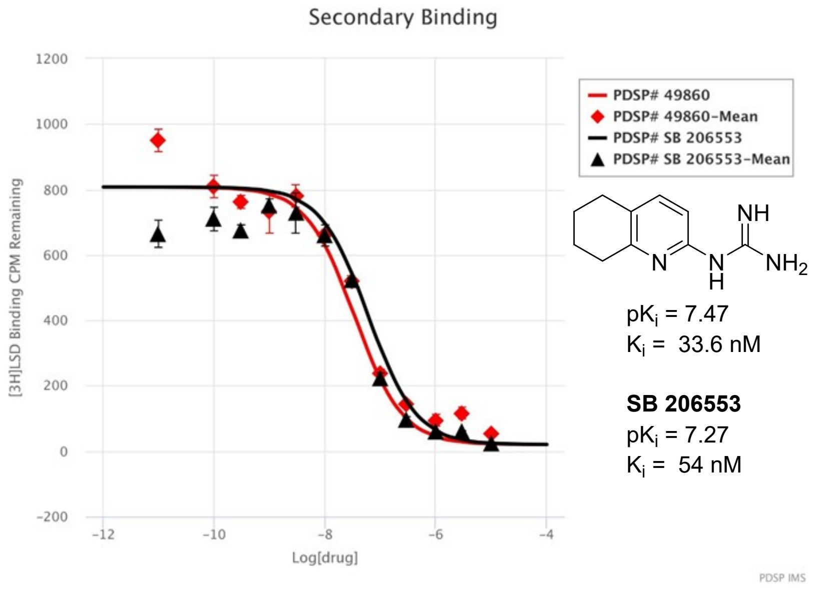

Figure S6. Competition binding isotherm for **1** with the 5-HT_{2B} receptor (reference: SB-206553; "hot ligand": [^{3}H]-LSD).

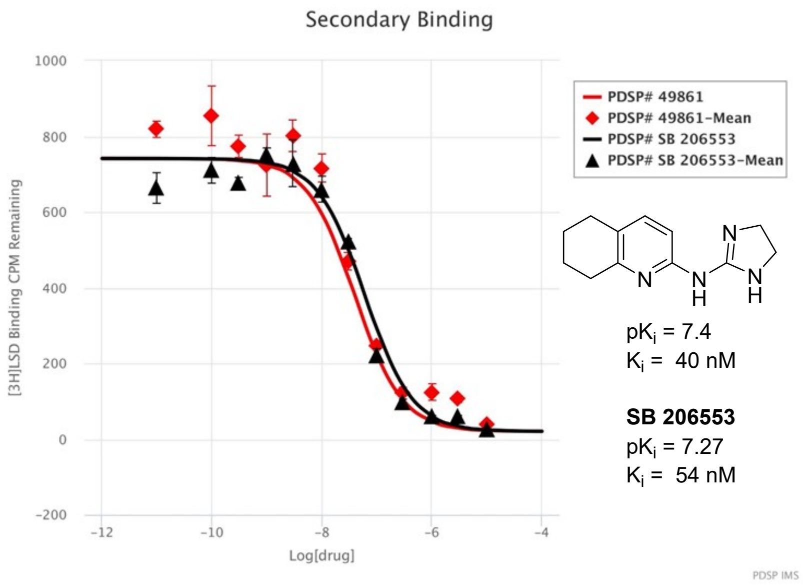

Figure S7. Competition binding isotherm for **3** with the 5-HT_{2B} receptor (reference: SB-206553; "hot ligand": [^{3}H]-LSD).

3. Effect of compounds 3, 4 and 6 on platelet aggregation induced by collagen in PRP.

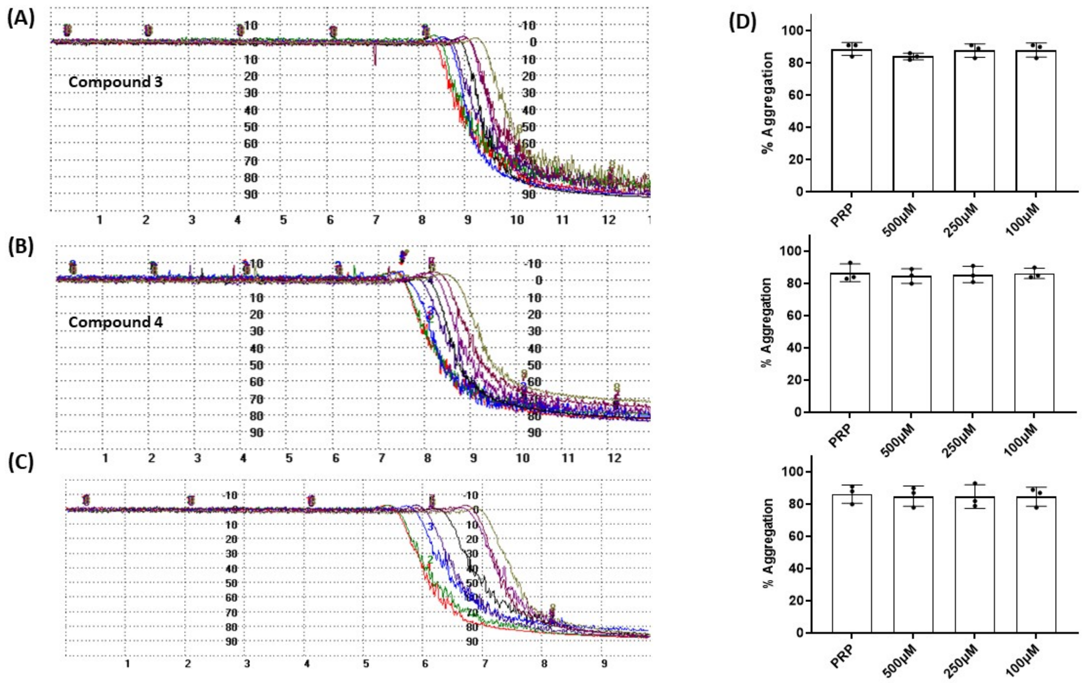

Figure S8. Effect of **3**, **4** and **6** on platelet aggregation induced by collagen (2 μg/mL). Representative traces from light transmission aggregometry showing the effect of (A) **3**, (B) **4** and (C) **6** on collagen-induced platelet aggregation at various concentrations. Untreated PRP stimulated by collagen was used as control. (D) Statistical analysis of (A), (B) and (C). Data is presented as mean ± SD. One way ANOVA $p>0.05$ ($n = 3$)

4. Guanidine derivatives 1, 2 and 5 do not have cytotoxic effects on platelets

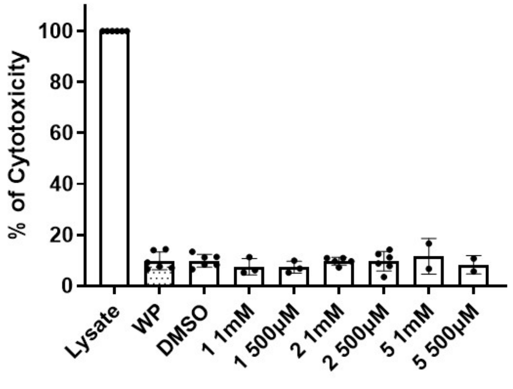

Figure S9. Statistical analysis of the toxic effects of **1, 2** and **5** (500 µM and 1 mM) in human platelets. Bars represent mean ± SD. One way ANOVA $p=0.9929$ ($n \geq 2$). Lysate, washed platelets (WP) and DMSO (vehicle) were used as controls.

5. Effect of compounds 1, 2 and 5 on the expression of PAC-1 and P-Selectin in platelet activation induced by collagen

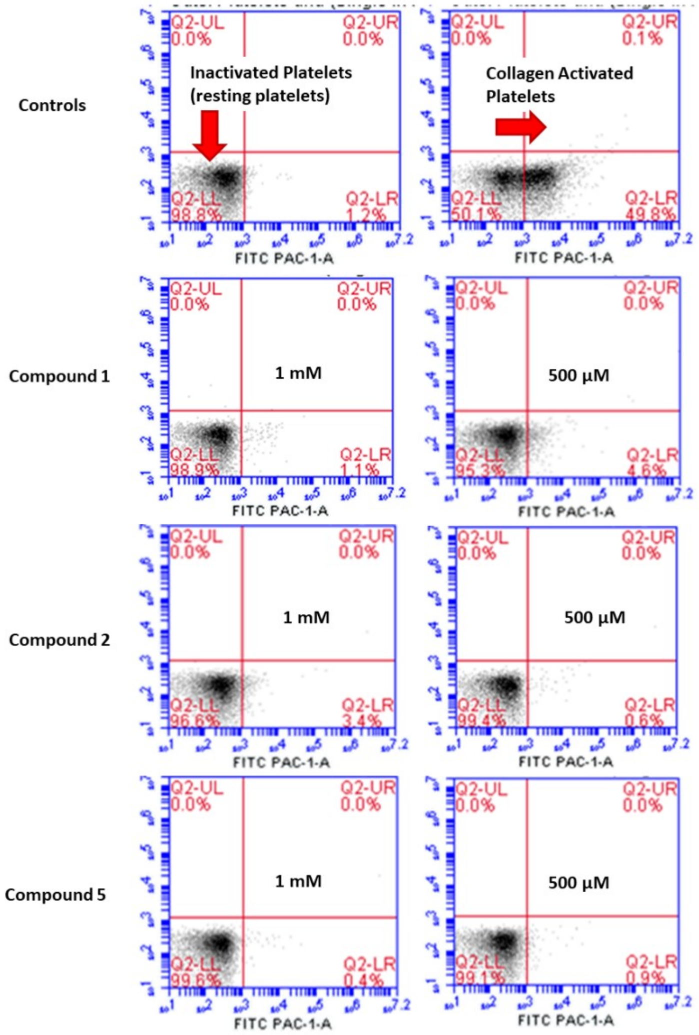

Figure S10. Representative plots from flow cytometry. PAC-1 expression on resting platelets, collagen stimulated platelets and collagen stimulated platelets incubated with compounds **1, 2** and **5** (1 mM and 500 μM).

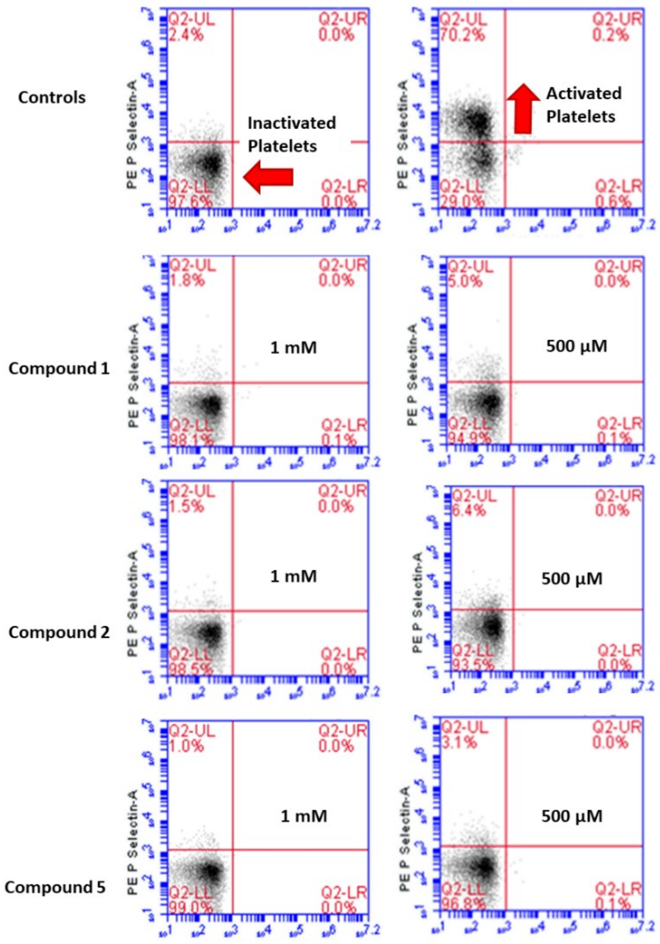

Figure S11. Representative plots from flow cytometry. P-Selectin expression on resting platelets, collagen stimulated platelets and collagen stimulated platelets incubated with compounds **1, 2** and **5** (1 mM and 500 µM).

References

1. B. Kelly, D. H. O'Donovan, J. O'Brien, T. McCabe, F. Blanco and I. Rozas, *J. Org. Chem.*, 2011, **76**, 9216–9227
2. F. Rodriguez, I. Rozas, J. E. Ortega, A. M. Erdozain, J. J. Meana and L. F. Callado, *J. Med. Chem.*, 2008, **51**, 3304–3312.
3. B. Kelly, M. McMullan, C. Muguruza, J. E. Ortega, J. J. Meana, L. F. Callado and I. Rozas, *J. Med. Chem.*, 2015, **58**, 963–977.
4. F. Rodriguez, I. Rozas, J. E. Ortega, J. J. Meana and L. F. Callado, *J. Med. Chem.*, 2007, **50**, 4516–4527.
5. B.L. Roth, *NIMH PDSP ASSAY Protoc. B.* 2018.
6. J. Besnard, et al., *Automated design of ligands to polypharmacological profiles.* Nature, 2012. **492**(7428): p. 215-20.
7. S. Kapur, and D. Mamo, *Half a century of antipsychotics and still a central role for dopamine D2 receptors.* Prog Neuropsychopharmacol Biol Psychiatry, 2003. **27**(7): p. 1081-90.
